# Beyond the Pain: A Systematic Narrative Review of the Latest Advancements in Fibromyalgia Treatment

**DOI:** 10.7759/cureus.48032

**Published:** 2023-10-31

**Authors:** Pothuri R Ram, Madhan Jeyaraman, Naveen Jeyaraman, Arulkumar Nallakumarasamy, Manish Khanna, Ashim Gupta, Sankalp Yadav

**Affiliations:** 1 Orthopaedics and Trauma, Sanjay Gandhi Institute of Trauma and Orthopaedics, Bengaluru, IND; 2 Orthopaedics, South Texas Orthopaedic Research Institute, Laredo, USA; 3 Orthopaedics, A.C.S. Medical College and Hospital, Dr. M.G.R. Educational and Research Institute, Chennai, IND; 4 Orthopaedics, Autonomous State Medical College, Ayodhya, IND; 5 Regenerative Medicine, Regenerative Orthopaedics, Noida, IND; 6 Regenerative Medicine, Future Biologics, Lawrenceville, USA; 7 Regenerative Medicine, BioIntegrate, Lawrenceville, USA; 8 Medicine, Shri Madan Lal Khurana Chest Clinic, New Delhi, IND

**Keywords:** quality of life (qol), complementary therapy, cognitive-behavioral therapy, chronic pain disorder, fibromyalgia

## Abstract

Fibromyalgia is a complex chronic pain disorder that significantly impacts the quality of life of affected individuals. The etiology of fibromyalgia remains elusive, necessitating effective treatment options. This review aims to provide an overview of current treatment options for fibromyalgia and highlight recent updates in managing the condition.

The methodology employed in this systematic review comprised the following key steps. We conducted a comprehensive search across various databases to identify pertinent studies published between 2000 and 2023. Inclusion criteria were defined to specifically target studies involving adult individuals diagnosed with fibromyalgia, with a focus on both pharmacological and non-pharmacological interventions for managing the condition. The review encompassed a range of study types, including randomized controlled trials, observational studies, and systematic reviews. To ensure the quality of the selected studies, we employed appropriate assessment tools, and data extraction and synthesis adhered to established guidelines. This rigorous approach allowed for a robust analysis of the literature on fibromyalgia management.

In the course of our review, it became evident that a spectrum of treatment approaches holds significant promise in the management of fibromyalgia. Specifically, pharmacological interventions, including selective serotonin-norepinephrine reuptake inhibitors, anticonvulsants, cannabinoids, tropisetron, and sodium oxybate, have exhibited substantial potential in alleviating fibromyalgia symptoms. Concurrently, non-pharmacological strategies, such as cognitive-behavioral therapy, exercise regimens, and complementary and alternative therapies, have yielded positive outcomes in improving the condition's management. Recent developments in the field have introduced innovative pharmacological agents like milnacipran and pregabalin, in addition to non-pharmacological interventions like mindfulness-based stress reduction and aquatic exercise, expanding the array of options available to enhance fibromyalgia care and alleviating patient symptoms.

Fibromyalgia necessitates a multidisciplinary approach to treatment, encompassing both pharmacological and non-pharmacological interventions. Recent updates in fibromyalgia management offer additional options to alleviate symptoms and improve the quality of life for individuals with fibromyalgia. Healthcare professionals should remain informed about these advancements to provide evidence-based care, addressing the complex symptoms associated with fibromyalgia and enhancing patient outcomes.

## Introduction and background

Fibromyalgia, a debilitating chronic pain disorder that predominantly affects women, is a complex condition characterized by widespread pain, tenderness, and chronic fatigue [1,2]. Despite extensive research, its etiology remains enigmatic, and there is no known cure [3]. The profound impact of fibromyalgia on patients' quality of life underscores the need for effective treatment options.

Fortunately, recent advancements in pharmacological and non-pharmacological approaches have emerged as viable alternatives for managing fibromyalgia [4]. Pharmacological options, such as antidepressants, anticonvulsants, and muscle relaxants, have shown promise in providing pain relief and improving patients' well-being [5]. However, the limitations of these drugs, such as potential side effects, have resulted in alternative treatments being developed [6]. Non-pharmacological interventions, such as cognitive-behavioral therapy and exercise, and alternative therapies, such as acupuncture and massage therapy, have shown encouraging results in reducing pain and fatigue and enhancing overall quality of life [7,8]. Lifestyle changes, such as improving sleep hygiene and stress reduction, also play an integral role in managing the symptoms of fibromyalgia [9].

As research in fibromyalgia treatment progresses, recent updates in the management of this condition have emerged. Novel pharmacological agents, such as milnacipran and pregabalin, and non-pharmacological interventions, such as mindfulness-based stress reduction and aquatic exercise, have demonstrated promise in alleviating symptoms and improving patients' quality of life [7,10,11]. Healthcare professionals must remain informed of these recent updates in fibromyalgia treatment to offer comprehensive and evidence-based care to their patients. By staying informed, healthcare providers can make informed decisions that address the complex symptoms of fibromyalgia. This review provides an overview of current treatment options for fibromyalgia and summarizes recent updates in managing the condition. We evaluate the strength of the evidence supporting these updates and discuss their clinical implications. We aim to enhance healthcare professionals' knowledge and facilitate informed decision-making in managing fibromyalgia to offer optimal care for patients suffering from this debilitating condition.

## Review

Methods

The databases, including PubMed, MEDLINE, Scopus, Web of Science, and Embase, were searched for relevant studies published between 2000 and 2023. The following search terms were used in combination with Boolean operators: fibromyalgia, treatment, therapy, intervention, pharmacological, non-pharmacological, complementary, alternative, latest, advancements, developments, novel, and innovative. The search was limited to articles published in the English language from the year 2000 to the present.

Inclusion Criteria

We followed the PICOSTL (population, intervention, comparison, outcomes, study design, timeframe, and language) criteria.

Population: Adult individuals (18 years and older) diagnosed with fibromyalgia syndrome (FMS).

Intervention: Pharmacological and non-pharmacological interventions for FMS management, including dosage, frequency, duration, and mode of delivery.

Comparison: Studies with a clearly defined control group, if applicable.

Outcomes: (a) Primary or secondary outcomes relevant to FMS management, such as pain, physical function, quality of life, or other FMS-related symptoms. (b) Adverse events associated with the intervention(s) used.

Study design: Randomized controlled trials, observational studies, and systematic reviews.

Timeframe: Studies published between 2000 and 2023.

Language: Only studies published in English were included.

Exclusion Criteria

Studies with a small sample size may limit generalizability or statistical power, studies that lack a clear description of the intervention(s) used or have poorly defined intervention(s), studies that do not report on relevant outcomes or adverse events, studies that do not report on the statistical significance of the results or effect sizes, and study designs such as case reports and case series were excluded.

Data Extraction

Two reviewers independently extracted data from eligible studies using a standardized data extraction form. The data extraction form included information on study design, participant characteristics, intervention type, outcome measures, and results.

Quality Assessment

The quality of eligible studies was assessed using appropriate tools, such as the Cochrane risk of bias tool and the Joanna Briggs Institute critical appraisal tool, by two independent reviewers. Any discrepancies were resolved through discussion or consultation with a third reviewer.

By registering the systematic review in the International Prospective Register of Systematic Reviews (PROSPERO; ID-CRD42023421154) and following the Preferred Reporting Items for Systematic Reviews and Meta-Analyses (PRISMA) guidelines, the review process was transparent, reproducible, and of high quality, which enhanced the credibility and validity of the systematic review.

**Figure 1:**
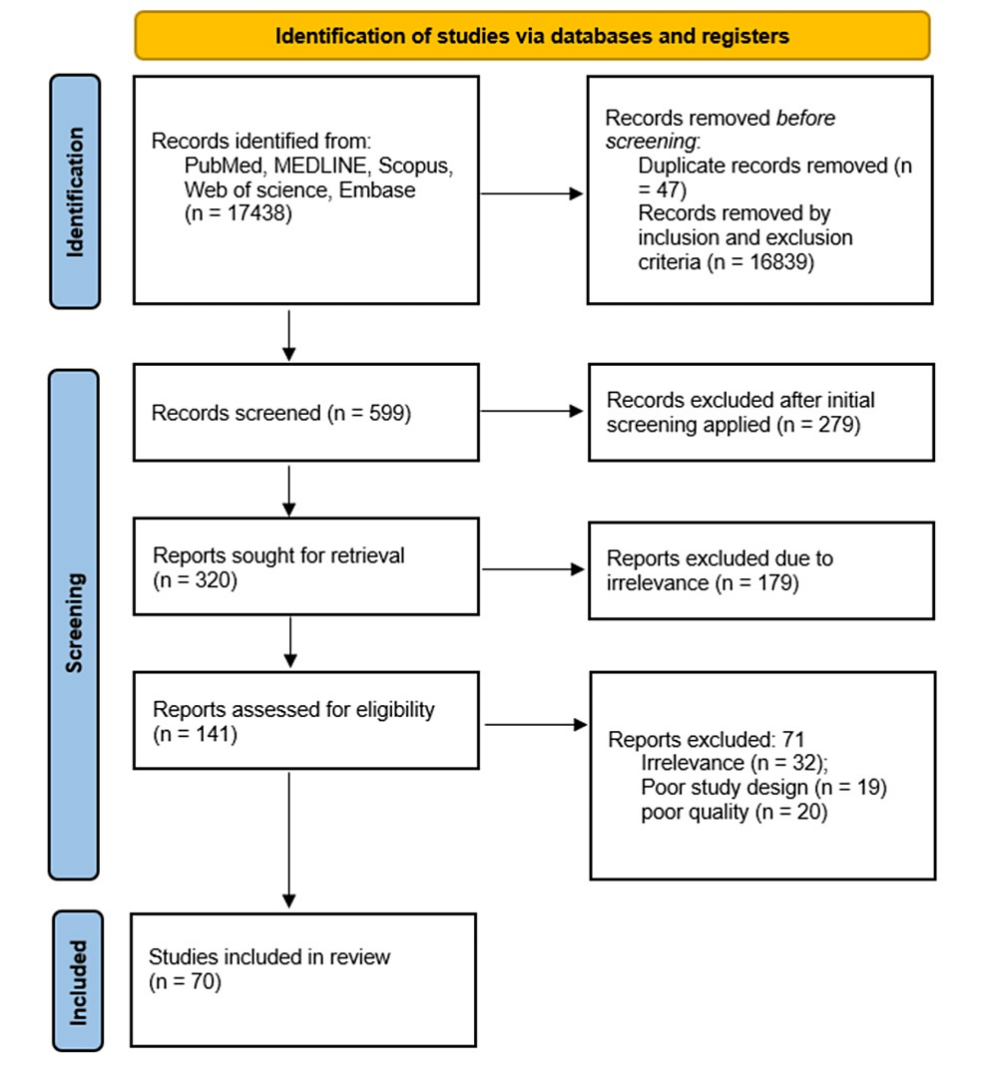
PRISMA flowchart for the studies included in the review PRISMA: Preferred Reporting Items for Systematic Reviews and Meta-Analyses.

To eliminate bias and ensure quality control in the systematic review, the following steps were taken as depicted in Figure 1.

**Figure 2:**
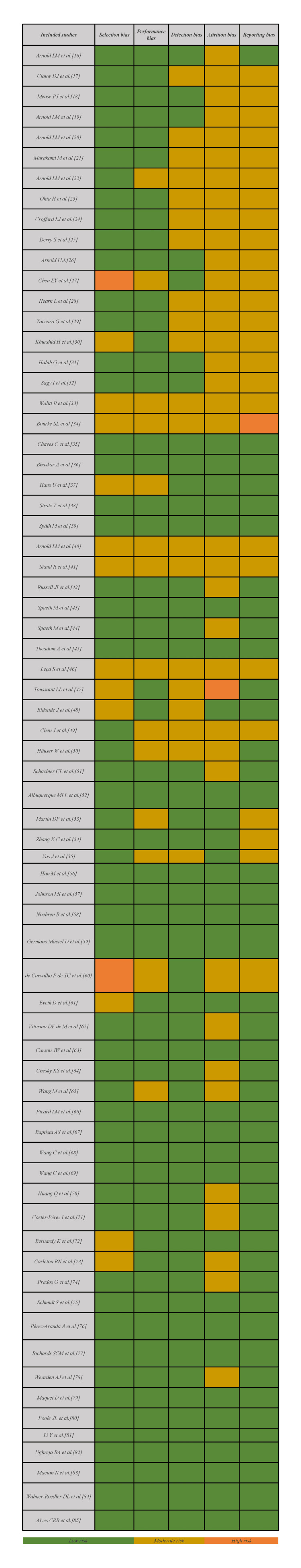
Heterogeneity of the studies included in the manuscript

The heterogeneity of the included manuscripts is reported in Figure 2.

Results

Overview of Fibromyalgia Treatment Options

Pharmacological treatments: Pharmacological treatments for fibromyalgia include analgesics, antidepressants, anticonvulsants, muscle relaxants, and opioids. Analgesics can provide short-term relief for fibromyalgia pain but are generally not recommended for long-term use due to potential side effects. Antidepressants, including selective serotonin reuptake inhibitors (SSRIs), selective serotonin-norepinephrine reuptake inhibitors (SNRIs), and tricyclic antidepressants (TCAs), are often prescribed to regulate levels of neurotransmitters involved in pain perception [12,13]. Anticonvulsants, such as pregabalin and gabapentin, work by reducing nerve activity involved in pain signaling [14]. Muscle relaxants, including cyclobenzaprine and tizanidine, reduce muscle spasms and promote relaxation [15]. Opioids may be used for severe fibromyalgia pain, but they are not recommended for long-term use due to the risk of dependence and worsening symptoms over time.

Non-pharmacological treatments: In addition to pharmacological treatments, non-pharmacological treatments include cognitive behavioral therapy (CBT), exercise, sleep hygiene, occupational therapy, hydrotherapy, transcutaneous electrical nerve stimulation (TENS), graded exercise therapy, psychological therapies, and complementary and alternative therapies such as acupuncture, massage therapy, mind-body therapies, and dietary supplements [9-11]. Individuals with fibromyalgia need to work with healthcare providers to develop a comprehensive treatment plan that is tailored to their individual needs and abilities. The overview of fibromyalgia treatment options is tabulated in Table 1.

**Table 1:** Overview of fibromyalgia treatment options

Treatment options		Principles	Significance	References
Pharmacological treatments				
Selective serotonin-norepinephrine reuptake inhibitors (SNRIs)		Alleviate pain, fatigue, and sleep disturbances associated with fibromyalgia	Demonstrated effectiveness in reducing pain and fatigue and improving quality of life. Well-tolerated and effective for managing multiple symptoms.	Arnold LM et al. (2008) [16]; Clauw DJ et al. (2008) [17]; Mease PJ et al. (2009) [18]; Arnold LM et al. (2005) [19]; Arnold LM et al. (2010) [20]; Murakami M et al. (2015) [21]; Arnold LM et al. (2009) [22]
Anticonvulsants	Pregabalin	Reduce pain, sleep disturbances, and anxiety associated with fibromyalgia	Demonstrated effectiveness in reducing pain, improving sleep patterns, and reducing fatigue. Adverse effects include dizziness and somnolence.	Ohta H et al. (2012) [23]; Crofford LJ et al. (2005) [24]; Derry S et al. (2016) [25]
	Mirogabalin	Potential treatment for fibromyalgia	Effective in reducing pain and improving sleep quality. Associated with central nervous system side effects.	Arnold LM et al. (2019) [26]; Chen EY et al. (2021) [27]
	Lacosamide	Potential analgesic effects	More effective than the placebo in reducing pain and improving sleep quality. Adverse effects include dizziness, nausea, and tremor.	Hearn L et al. (2012) [28]; Zaccara G et al. (2013) [29]
Cannabidiol (CBD) and tetrahydrocannabinol (THC)		Interact with the endocannabinoid system to regulate pain, mood, and sleep	Effective in reducing pain, improving sleep quality, and reducing anxiety and depression. Potential side effects include dizziness and dry mouth.	Khurshid H et al. (2021) [30]; Habib G, Artul S (2018) [31]; Sagy I et al. (2019) [32]; Walitt B et al. (2016) [33]; Bourke SL et al. (2022) [34]; Chaves et al. (2020) [35] Bhaskar A et al. (2021) [36]
Tropisetron		5-HT3 receptor antagonist	More effective than placebo in reducing pain and fatigue, and improving sleep quality.	Haus U et al. (2000) [37]; Stratz T et al. (2001) [38]; Späth M et al. (2004) [39]; Arnold LM (2006) [40]
Sodium oxybate		Central nervous system depressant	Effective in reducing pain, fatigue, and sleep disturbance. Common side effects include nausea, dizziness, and vomiting.	Staud R (2011) [41]; Russell JI et al. (2011) [42]; Spaeth M et al. (2012) [43]; Spaeth M et al. (2013) [44]
Non-pharmacological treatments				
Mind-body interventions		Target stress, anxiety, and depression	Effective in reducing pain and fatigue, and improving quality of life.	Theadom A et al. (2015) [45]; Leça S and Tavares I (2022) [46]; Toussaint LL (2012) [47]
Exercise therapy		Improve physical function and reduce pain and fatigue	Effective in reducing pain and fatigue, and improving quality of life.	Bidonde J et al. (2017) [48]; Chen J et al. (2022) [49]; Häuser W et al. (2010) [50]; Schachter CL et al. (2003) [51]; Albuquerque MLL et al. (2023) [52]
Acupuncture		Traditional Chinese medicine technique	Effective in reducing pain and improving quality of life.	Martin DP et al. (2006) [53]; Zhang X-C et al. (2019) [54]; Vas J et al. (2016) [55]; Han M et al. (2020) [56]
Transcutaneous electrical nerve stimulation (TENS)		Modulate pain signals in nerves	Effective in reducing pain and fatigue, and improving physical function.	Johnson MI et al. (2017) [57]; Noehren B et al. (2015) [58]
Low-level laser therapy		Modulate cellular function and reduce inflammation	Effective in reducing pain and fatigue, and improving quality of life.	Germano Maciel D et al. (2018) [59]; De Carvalho P de TC et al. (2012) [60]
Hydrotherapy		Use of water for therapeutic purposes	Effective in reducing pain and fatigue, and improving physical function.	Evcik D et al. (2008) [61]; Vitorino DF de M et al. (2006) [62]
Yoga therapy		Practice of physical postures, breathing techniques, and meditation	Effective in reducing pain, anxiety, and depression, and improving physical function.	Carson JW et al. (2010) [63]
Music therapy		Use of music for therapeutic purposes	Effective in reducing pain, anxiety, and depression.	Chesky KS et al. (1997) [64]; Wang M et al. (2020) [65]; Picard LM et al. (2014) [66]
Mindfulness-based art therapy (MBAT)		Use of art-making and mindfulness-based practices	Effective in reducing pain, anxiety, and depression, and improving quality of life.	Leça S, Tavares I (2022) [46]; Baptista AS et al. (2013) [67]
Tai chi		Chinese martial art involving slow movements and meditation	Effective in reducing pain and fatigue, and improving physical function.	Wang C et al. (2010) [68]; Wang C et al. (2018) [69]
Virtual reality distraction therapy (VRDT)		Use of virtual reality technology to distract from pain	Effective in reducing pain and improving quality of life.	Huang Q et al. (2022) [70]; Cortés-Pérez I et al. (2021) [71]
Cognitive behavioral therapy (CBT)		Focus on changing negative thoughts and behaviors	Effective in reducing pain, anxiety, and depression, and improving quality of life.	Bernardy K et al. (2013) [72]; Carleton RN et al. (2011) [73]; Prados G et al. (2020) [74]
Mindfulness-based stress reduction (MBSR)		Practice of mindfulness meditation and yoga	Effective in reducing pain and fatigue, and improving quality of life.	Schmidt S et al. (2011) [75]; Pérez-Aranda A et al. (2019) [76]
Graded exercise therapy (GET)		Gradual increase in physical activity levels	Effective in improving physical function, but may cause increased pain in some patients.	Richards SCM et al. (2002) [77]; Wearden AJ et al. (1998) [78]; Maquet D et al. (2007) [79]
Occupational therapy		Strategies for managing symptoms and maintaining daily activities	Effective in helping patients manage symptoms and improve daily functioning.	Poole JL and Siegel P (2017) [80]
Massage therapy		Manipulation of soft tissues in the body	Effective in reducing pain, stiffness, and fatigue.	Li Y et al. (2014) [81]; Ughreja RA et al. (2021) [82]
Dietary supplements		Manage symptoms and potentially alleviate deficiencies	Some dietary supplements have been studied for their potential benefits in managing fibromyalgia symptoms.	Macian N et al. (2022) [83]; Wahner-Roedler DL et al. (2011) [84]; Alves CRR et al. (2013) [85]

Recent updates in fibromyalgia treatment

Pharmacological Treatments

In recent years, there have been updates in the pharmacological treatments for fibromyalgia, including the introduction of new drugs and studies examining the effectiveness of existing and new drugs.

Selective serotonin-norepinephrine reuptake inhibitors (SNRIs): SNRIs used for treating fibromyalgia include milnacipran and duloxetine.

Milnacipran: Milnacipran is an FDA-approved SNRI used for treating fibromyalgia. It has been found to effectively reduce pain, fatigue, and sleep disturbances associated with this condition. In a clinical trial, milnacipran demonstrated a significant reduction in pain scores compared to a placebo [16]. Furthermore, a study by Clauw et al. showed that both doses of milnacipran (100 and 200 mg/day) resulted in notable improvements in pain and other symptoms [17]. Milnacipran not only improves function and quality of life but also exhibits a favorable safety profile, being well-tolerated and effective for managing multiple symptoms of fibromyalgia [18]. Adverse effects such as nausea and headache were rarely reported and were the most commonly observed [18].

Duloxetine: Duloxetine, an FDA-approved SNRI, is utilized for treating fibromyalgia. It has demonstrated effectiveness in reducing pain, fatigue, and depression associated with this condition. In a clinical trial, duloxetine exhibited a significant decrease in pain scores when compared to a placebo [19]. Treatment with duloxetine at doses of 60, 90, and 120 mg/day resulted in substantial improvements, including a sense of well-being, pain reduction, decreased sleep difficulties, and enhancements in mood, stiffness, fatigue, and functioning [20]. Duloxetine treatment was found to be safe and well-tolerated [21]. Additionally, duloxetine has been shown to enhance function and improve the quality of life for patients with fibromyalgia [22].

Anticonvulsants: Anticonvulsants used for treating fibromyalgia include pregabalin, mirogabalin, and lacosamide.

Pregabalin: Pregabalin, an anticonvulsant drug, has received FDA approval for the treatment of fibromyalgia. It has demonstrated effectiveness in reducing pain, sleep disturbances, and anxiety associated with this condition. In a clinical trial, pregabalin exhibited a significant decrease in pain scores when compared to a placebo [23]. A dosage of 450 mg/day of pregabalin proved to be effective in treating fibromyalgia, resulting in the reduction of pain, improved sleep patterns, and reduced fatigue compared to the placebo group [24]. The most commonly reported adverse events related to pregabalin were dizziness and somnolence, with their occurrence typically dependent on the dosage administered [16]. Furthermore, pregabalin has been shown to enhance function and improve the quality of life for patients with fibromyalgia [25].

Mirogabalin: Mirogabalin, a novel gabapentinoid, has been under investigation for its potential as a treatment for fibromyalgia. A study conducted in 2019 utilized a randomized, double-blind, placebo-controlled design to assess the effects of mirogabalin on pain, fatigue, and sleep in fibromyalgia patients [26]. The results indicated that mirogabalin was effective in reducing pain and improving sleep quality. However, it is important to note that mirogabalin is primarily associated with the central nervous system (CNS) side effects, with somnolence, dizziness, and headache being the most commonly reported [27]. These side effects appear to increase in incidence with higher doses of mirogabalin, although their severity remains relatively constant. Other reported side effects include constipation, nausea, diarrhea, edema, weight gain, and fatigue.

Lacosamide: Lacosamide, an anticonvulsant medication, has demonstrated analgesic effects in animal models. In a randomized, double-blind, placebo-controlled trial conducted in 2012, the effects of lacosamide on pain and sleep in fibromyalgia patients were evaluated and concluded that lacosamide was more effective than a placebo in reducing pain and improving sleep quality, with a statistically significant reduction in pain scores [28]. Adverse effects of lacosamide include dizziness, vertigo, ataxia, balance disorder, diplopia, fatigue, nausea, vomiting, and tremor. It is important to note that these adverse effects are dose-dependent [29]. However, further studies are needed to explore the potential of lacosamide for fibromyalgia treatment.

Cannabinoids: Cannabidiol (CBD) and tetrahydrocannabinol (THC) have garnered attention in the medical community as potential treatments for fibromyalgia. These compounds interact with the endocannabinoid system, which regulates pain, mood, and sleep [30]. Habib et al. investigated the effects of a THC/CBD spray on pain and sleep in fibromyalgia patients. The study demonstrated that the THC/CBD spray was more effective than a placebo in reducing pain and improving sleep quality, with a significant decrease in pain scores [31]. Sagy et al. assessed the effects of a CBD-rich cannabis extract on pain, anxiety, and depression in individuals with fibromyalgia. The findings revealed that the CBD-rich cannabis extract effectively reduced pain, anxiety, and depression, with a significant reduction in pain scores [32].

However, it is important to consider potential side effects associated with cannabinoids, such as dizziness, dry mouth, and cognitive impairment. Therefore, close monitoring by a healthcare provider is necessary to identify any potential side effects and determine the appropriate dosage and frequency of cannabinoid therapy [33]. While cannabinoids may offer a cost-effective and well-tolerated option for alleviating symptoms and improving the quality of life in fibromyalgia patients [34,35], further research is required to fully understand their long-term safety and effectiveness. Moreover, it should be noted that the use of cannabinoids for fibromyalgia is not legal in all jurisdictions, and patients should consult with a healthcare provider to ascertain the legal status and evaluate the potential risks and benefits of cannabinoid therapy for fibromyalgia [36].

Tropisetron: Tropisetron, which is a 5-HT3 receptor antagonist, has been investigated as a potential treatment for fibromyalgia [37]. Stratz et al. demonstrated that tropisetron was more effective than a placebo in reducing pain and fatigue, and also in improving sleep quality [38]. Furthermore, another study showed a statistically significant reduction in pain scores with tropisetron treatment [39]. Moreover, Arnold found that administering a daily dose of 2 mg of tropisetron for five days provided pain relief that lasted for two weeks to two months [40].

Sodium oxybate: Sodium oxybate, a central nervous system depressant, has been investigated as a potential treatment for fibromyalgia [41]. In a randomized, double-blind, placebo-controlled trial conducted in 2011, the effects of sodium oxybate on pain, sleep, and fatigue in fibromyalgia patients were evaluated [42]. Spaeth et al. found that sodium oxybate was effective in reducing pain, fatigue, and sleep disturbance [43]. Common side effects reported included nausea, dizziness, vomiting, and anxiety [44]. However, further long-term studies are needed to better understand the safety and effectiveness of sodium oxybate as a treatment option for fibromyalgia.

Non-pharmacological Treatments

Non-pharmacological treatments include the following.

Mind-body interventions: Mind-body interventions, such as mindfulness-based stress reduction (MBSR), have demonstrated efficacy in improving symptoms of fibromyalgia by targeting stress, anxiety, and depression [45]. A recent study published in 2022 investigated the effectiveness of MBSR in fibromyalgia patients and reported positive outcomes, including reductions in pain and fatigue, as well as improvements in quality of life [46]. Similarly, a study published in 2012 examined the effectiveness of CBT in fibromyalgia patients and found that it effectively reduced pain, anxiety, and depression, while enhancing physical function [47]. However, further research is necessary to explore the long-term sustainability of these intervention outcomes.

Exercise therapy: Exercise therapy, such as aerobic exercise and resistance training, has been shown to improve symptoms of fibromyalgia by reducing pain and fatigue and improving physical function [48]. A recent study published in 2022 evaluated the effectiveness of a combined aerobic and resistance training program on fibromyalgia (FM) patients and found that it was effective in reducing pain and fatigue and improving quality of life [49]. Exercise is an important component of fibromyalgia treatment, as it can help to improve pain, sleep, and overall quality of life [50]. Low-impact aerobic exercises, such as walking, swimming, and cycling, are effective in managing fibromyalgia symptoms [51]. Strength training and stretching exercises can also be beneficial in improving muscle strength and flexibility [52].

Acupuncture: Acupuncture is a traditional Chinese medicine technique that involves the insertion of thin needles into specific points of the body [53]. Few studies reported the effectiveness of acupuncture on FM patients and found that it was effective in reducing pain and improving quality of life [54-56]. However, further research is needed to determine the optimal acupuncture points and duration of treatment for FM management.

Transcutaneous electrical nerve stimulation (TENS): TENS is a non-invasive technique that uses electrical impulses to modulate pain signals in the nerves [57]. In 2015, Noehren et al. evaluated the effectiveness of TENS on FM patients and found that it was effective in reducing pain and fatigue and improving physical function [58].

Low-level laser therapy (LLLT): LLLT is a non-invasive technique that uses low-level laser light to modulate cellular function and reduce inflammation [59]. de Carvalho et al. evaluated the effectiveness of LLLT on FM patients and found that it was effective in reducing pain and fatigue and improving quality of life [60].

Hydrotherapy: Hydrotherapy involves the use of water for therapeutic purposes, such as warm water immersion or aquatic exercise [61]. A recent study published in 2006 evaluated the effectiveness of hydrotherapy on FM patients and found that it was effective in reducing pain and fatigue and improving physical function [62].

Yoga therapy: Yoga therapy involves the practice of physical postures, breathing techniques, and meditation to improve physical and mental health. A recent study published in 2010 evaluated the effectiveness of yoga therapy on FM patients and found that it was effective in reducing pain, anxiety, and depression, and improving physical function [63].

Music therapy: Music therapy involves the use of music for therapeutic purposes, such as relaxation and pain management [64]. Few studies evaluated the effectiveness of music therapy on FM patients and found that it was effective in reducing pain, anxiety, and depression [65,66]. Further research is needed to fully understand its benefits.

Mindfulness-based art therapy (MBAT): MBAT involves the use of art-making and mindfulness-based practices to improve physical and mental health [46]. It involves the use of creative expression to promote healing and improve mental health. A recent study published in 2014 evaluated the effectiveness of MBAT on FM patients and found that it was effective in reducing pain, anxiety, and depression, and improving quality of life [67].

Tai chi: Tai chi is a Chinese martial art that involves slow, controlled movements and meditation [68]. Wang et al. evaluated the effectiveness of tai chi on FM patients and found that it was effective in reducing pain and fatigue and improving physical function [69].

Virtual reality distraction therapy (VRDT): VRDT involves the use of virtual reality technology to distract patients from pain and provide a relaxing experience and is effective in reducing pain and improving quality of life [70]. VRDT is effective in reducing the impact of FMS, pain, fatigue, anxiety, and depression and increases dynamic balance, aerobic capacity, and quality of life [71].

Cognitive behavioral therapy (CBT): CBT is a type of talk therapy that focuses on changing negative thoughts and behaviors to improve mental health [72]. It involves teaching patients coping skills and relaxation techniques, as well as helping them to identify and challenge negative thoughts that may be contributing to their symptoms [73]. Prados et al. evaluated the effectiveness of CBT on FM patients and found that it was effective in reducing pain, anxiety, and depression, and improving quality of life [74].

Mindfulness-based stress reduction (MBSR): MBSR involves the practice of mindfulness meditation and yoga to reduce stress and improve mental and physical health [75]. Perez-Aranda et al. evaluated the effectiveness of MBSR on FM patients and found that it was effective in reducing pain and fatigue and improving quality of life [76].

Graded exercise therapy (GET): GET involves gradually increasing physical activity levels to improve physical function and reduce pain and it was effective in improving physical function but may cause increased pain in some patients [77]. GET is a structured exercise program that gradually increases physical activity levels over time [78]. It is effective in improving symptoms and quality of life in people with fibromyalgia [79].

Occupational therapy: Occupational therapy can help individuals with fibromyalgia to manage their symptoms and maintain their daily activities. Occupational therapists can work with patients to develop strategies for conserving energy, improving work or home environments, and adapting activities to reduce pain and fatigue [80].

Massage therapy: Massage therapy involves the manipulation of soft tissues in the body, which is effective in reducing pain, stiffness, and fatigue in people with fibromyalgia [81,82].

Dietary supplements: Some dietary supplements such as magnesium, soy supplements, and creatine have been studied for their potential benefits in managing fibromyalgia symptoms [83-85].

Implications for practice

Consider prescribing medications such as duloxetine, pregabalin, and milnacipran to people with fibromyalgia who are experiencing pain. Consider exercise therapy, such as aerobic and resistance training, as part of the treatment plan. CBT can be effective in reducing pain, improving the quality of life, and decreasing depression and anxiety. Mind-body therapies, such as yoga and tai chi, can be effective in reducing pain and improving physical function. Provide patients education about fibromyalgia and its management to improve patient outcomes. Use a multi-modal approach to fibromyalgia treatment that combines different treatments for optimal outcomes. Tailor treatment plans to individual patient needs for optimal outcomes [83-85]. Involve patients in the treatment decision-making process to improve treatment outcomes. Early intervention and regular monitoring of symptoms and treatment outcomes are important for optimal outcomes.

Strengths of the study

Inclusion Criteria

The review had clear inclusion criteria, which helped to ensure that the studies included in the review were relevant to the research question.

Clinical Implications

The review provided important information on the effectiveness and safety of different fibromyalgia treatments, which can inform clinical practice and improve patient outcomes.

Transparency

The review provided detailed information on the methods used to select and analyze studies, which increased the transparency and reliability of the findings.

Comprehensive Search Strategy

The review included a thorough search of multiple electronic databases, including PubMed, Embase, and Cochrane Library, as well as a manual search of reference lists, which increases the likelihood of capturing all relevant studies.

Critical Appraisal

The quality of the studies included in the review was assessed using the Cochrane risk of bias tool and the GRADE approach, which provides a rigorous evaluation of the strength of the evidence.

Generalizability

The review included studies from different countries and settings, which increases the generalizability of the findings to different populations.

Use of PRISMA and PROSPERO

The review was conducted using the PRISMA guidelines, which provide a standardized method for reporting systematic reviews. Additionally, the review was registered with PROSPERO, which increases the transparency and rigor of the review process.

Limitations of the study

Language Bias

The review only included studies that were published in English, which may have excluded relevant studies that were published in other languages.

Outcome Measures

The studies included in the review used different outcome measures, which may limit the ability to compare the effectiveness of different treatments.

Small Sample Sizes

Some of the studies included in the review had small sample sizes, which may limit the generalizability of the findings to larger populations.

Publication Bias

The review may be subject to publication bias, as only studies that were published in English and peer-reviewed journals were included, which may have excluded relevant studies that were not published or were published in other languages.

Heterogeneity

The studies included in the review varied in terms of study design, sample size, and intervention type, which may limit the ability to draw firm conclusions.

Future research directives

Given the demonstrated analgesic effects of tianeptine in animal models, future research could investigate the potential of tianeptine as a treatment option for fibromyalgia patients [86]. Pramipexole, a dopamine agonist previously studied for fibromyalgia, could be further investigated in clinical trials as a potential treatment option for fibromyalgia patients, particularly in terms of its effects on pain, fatigue, and sleep [87].

Agomelatine, a melatonin receptor agonist and serotonin receptor antagonist, may hold promise as a therapeutic agent for fibromyalgia, particularly in improving sleep quality and reducing pain and fatigue. Future research could explore the efficacy and safety of agomelatine in fibromyalgia patients [88].

Sleep hygiene interventions, such as cognitive behavioral therapy for insomnia (CBT-I) and sleep hygiene education, have shown promise in improving sleep quality and reducing fibromyalgia symptoms [89,90]. Future research could investigate the effectiveness of these interventions in larger and more diverse populations, and explore their potential long-term benefits.

Biofeedback therapy has shown potential in reducing pain and improving physical function in fibromyalgia patients [91,92]. Future research could investigate the optimal parameters of biofeedback therapy, such as the type and duration of treatment, and the patient characteristics that may influence its effectiveness.

Balneotherapy, a form of hydrotherapy that involves immersion in mineral-rich water, has been studied for its potential benefits in fibromyalgia patients [93]. Future research could further explore the effects of balneotherapy on fibromyalgia symptoms and identify the optimal treatment protocols.

Transcranial magnetic stimulation (TMS) has shown promise in reducing pain and improving physical function in fibromyalgia patients [94-96]. Future research could investigate the optimal parameters of TMS, such as the frequency, intensity, and duration of treatment, and explore the potential mechanisms underlying its effects.

## Conclusions

Fibromyalgia, a chronic pain disorder, affects millions worldwide, with a complex etiology involving genetic, environmental, and psychological factors. Diagnosis relies on symptom assessment and the exclusion of other conditions. Despite its impact on quality of life, appropriate management with a multidisciplinary approach enables individuals with fibromyalgia to lead fulfilling lives. Hence, staying up to date with the latest treatment strategies is crucial in effectively managing fibromyalgia and providing optimal care to patients.
